# Bayesian Hierarchical Modeling for Categorical Longitudinal Data from Sedation Measurements

**DOI:** 10.1155/2013/579214

**Published:** 2013-07-10

**Authors:** Erol Terzi, Mehmet Ali Cengiz

**Affiliations:** Department of Statistic, Ondokuz Mayis University, 55139 Samsun, Turkey

## Abstract

We investigate a Bayesian hierarchical model for the analysis of categorical longitudinal data from sedation measurement for Magnetic Resonance Imaging (MRI) and Computerized Tomography (CT). Data for each patient is observed at different time points within the time up to 60 min. A model for the sedation level of patients is developed by introducing, at the first stage of a hierarchical model, a multinomial model for the response, and then subsequent terms are introduced. To estimate the model, we use the Gibbs sampling given some appropriate prior distributions.

## 1. Introduction 

Magnetic Resonance Imaging (MRI) and Computerized Tomography (CT) require the patient to lie still for periods of up to 60 min. These two diagnostic procedures also require strict immobility and sedation for a successful result. If a child cannot remain adequately still for examination, sedation may be necessary. Optimal sedation management of children before MRI and CT has received attention in the last decade [1, 2]. The sedation medications must be chosen carefully for children's safety and effectiveness. Many researches related to the comparison of different sedation medications have been performed successfully [3, 4]. In these studies, for each medication group sedation levels were obtained at different time points within the time up to 60 min. In addition to sedation level measurements, the other multiple assessments of the same patient were recorded, and the within subject ones, such as sedation levels at different time points for a given patient, were correlated. This case is an example when a longitudinal study is made with responses being measured repeatedly on the same patient across time. In medical studies, statistical analysis of the data set described earlier has been performed by many researchers, who use the known methods such as ANOVA, MANOVA, and Linear Models, assuming that the repeated observations from each patient are uncorrelated. Since repeated observations are made on the same patient, observed responses are generally correlated. For robust analysis, this association must be accounted for. Weighted least squares model is used for repeated categorical data. This model works well for large sample size, no missing data, a small number of response variables, and discrete independent variables. Recent years have witnessed new statistical methods of analysing for data that do not meet these conditions.

 Mathematical models for multiple regression, linear models, and time series are generally useful where random variables are approximately normal and can be explained by some linear structure. However, data can be clearly nonnormal when they represent categorical or frequency observations. Generalized Linear Models (GLMs) offer convenient and highly applicable tools for these kinds of data. They allow for more general structures and more general distributions than linear regression and ANOVA. Nelder and Wedderburn developed the concept of GLMs [5], and an extensive treatment was given by [6]. With the introduction of GLM, a much more flexible instrument for statistical modeling was created. As special cases, they include multiple linear regression, logit and probit models for quanta responses, and log linear response models for counts. Introduced Generalized Estimating Equations (GEEs) [7] were developed to extend the GLM introduced by [5]. 

 Longitudinal researches are defined as studies in which the response of each patient is observed on two or more occasions. They are often used in medical and health research. The methods used for the analysis of longitudinal data differ from the traditional regression analysis such as multiple regressions. Longitudinal data sets consist of repeated observations of an patient and a set of covariates for each of many patients which may be fixed or which may be changed with time. Longitudinal data sets are defined by the fact that repeated observations for a patient are correlated [8]. Therefore the modeling of the correlation structure is required. When the response variable is normal, a large class of linear models is available for analysis. However, when the response variable is categorical, other methods must be considered. In recent years, considerable effort has gone into the development of statistical methods for the analysis of longitudinal categorical response data. While much of this effort has focused on methods for binary or Poisson data, relatively little attention has been given to nominal categorical data. 

 More generally, hierarchical models describe efficiently complex datasets incorporating correlation or including other properties in our model. Hence, when multivariate or repeated responses are observed, correlation can be incorporated in the model via a common “random” effect for all measurements referring to the same individual. This introduces a marginal correlation between repeated data, while interpretation is based on the conditional means. Therefore, given the random effects, the structure and the interpretation are similar to common generalized linear models. Accordingly, hierarchical models naturally appear, for example, when modeling spatiotemporal data in which correlation between time and space can be added by using common random effects on adjacent (in time or space) responses. Hierarchical models can also be used to imply a complicated marginal distribution but (at the same time) keep the conditional structure as simple as possible [9]. 

 Bayesian analyses of hierarchical linear models have been considered for at least forty years [10] and have remained a topic of theoretical and applied interest [11–14]. Reference [15] reviews much of the extensive literature in the course of comparing Bayesian and non-Bayesian inferences for hierarchical models. As part of their article, Browne and Draper consider some different prior distributions for variance parameters; here, we explore the principles of hierarchical prior distributions in the context of a specific class of models. Hierarchical (multilevel) models are central to modern Bayesian statistics for both conceptual and practical reasons. At a practical level, hierarchical models are flexible tools for combining information and partial pooling of inferences [16–18].

 In this study, we use a Bayesian approach to fit several hierarchical models of increasing complexity to assess the significance of both fixed and random effects on sedation levels and investigate a Bayesian hierarchical model for the analysis of categorical longitudinal data from sedation measurement for MRI and CT. A model for the sedation level of patients is developed by introducing, at the first stage of a hierarchical model, a multinomial model for the response and then subsequent terms are introduced. 

## 2. Material and Method

There are several methods that may be used to estimate the determinants of sedation levels with categories (1,…, 6). 

 First method we considered is the multinomial logit approach. The model

(1) $$\begin{array}{c} \text{Pr}\left(Y_{it}=\frac{j}{X_{it}}\right)=\frac{\exp\left(X_{it}\beta_{j}\right)}{\sum_{k=1}^{j}\exp\left(X_{it}\beta_{k}\right)}, \end{array}$$

where Pr(*Y*_*it*_ = *j*/*X*_*it*_) is the probability that patient *i* has outcome *j* at time *t* given covariates *X*_*it*_ of the patient at that time. 

 In our analysis the response has six levels (*j* = 1,…, 6). For identifiability, *j* = 1 is set as the reference category so that the parameters estimated from the multinomial logistic model are interpreted as the logarithm of the change in the odds of being outcome *j* relative to that of being outcome 2 for a one unit change in the corresponding explanatory variable at time *t*. 

 We investigate the relationship between sedation levels and both categorical and continuous explanatory variables by specifying a Bayesian hierarchical model for the multinomial response. We also include the lagged response variable in the model to assess the probability of transition between the times. Reference [19] considers a dynamic multinomial logit panel model with random effects to explain the labour market level of individuals in urban Mexico. The individual effects are assumed to be independent of the observed characteristics and to follow a multivariate normal distribution. We use a similar model for explaining the sedation levels of patients in which the selected covariates and sedation levels at the time of the previous time may influence the sedation levels of a patient. 

 Assume that patient *i* ( = 1,…, *N*) can be in any of *j* possible levels at time *t* ( = 1,…, *T*). In the first level of the Bayesian hierarchical model, the *y*_*it*._ = (*y*_*it*1_,…, *y*_*itj*_) are assumed to be distributed as multinomial random variables. The response takes the value *y*_*itj*_ ∈ {1,2, 3,4, 5,6}. The model may be written as

(2) $$\begin{array}{c} y_{it.}\sim \text{multinomial}\left(P_{it.},n_{it}\right), \end{array}$$

where *P*_*itj*_ is the probability of patient *i* being in level *j* at time *t*. 

 The second level of the model relates the probabilities *P*_*itj*_ to the regression effects, lagged effects, and random effects such that

(3) $$\begin{array}{c} P_{itj}=\frac{\mu_{itj}}{\sum_{j}^{}\mu_{itj}},\\ \log it\left(\mu_{itj}\right)=X_{it}^{T}\beta_{j}+Z_{it}^{T}\gamma_{j}+\alpha_{ij}, \end{array}$$

where *X*_*it*_ is a matrix of explanatory variables, *Z*_*it*_ is a matrix of lagged level variables, *β*_*j*_ and *γ*_*j*_ are vectors of parameters to be estimated, and *α*_*ij*_ is a random effect reflecting time constant unobserved heterogeneity.

 To identify the model, we choose first level of sedation to be reference level (*j* = 1) with *β*_1_, *γ*_1_, and *α*_*i*1_ set to 0. It follows that log⁡⁡(*μ*_*it*1_) = 0, and, hence,

(4) $$\begin{array}{c} \log\left(\frac{P_{itj}}{P_{it1}}\right)=\log\left(\frac{\mu_{itj}}{\mu_{it1}}\right)=\log\left(\mu_{itj}\right). \end{array}$$

So log⁡⁡(*μ*_*itj*_) can be interpreted as the log of the probability of being in level *j* relative to the probability of being in level 1. 

 The posterior distributions for all parameters are as follows

(5) P(β,γ,α,Σ ∣ y)∝P(y ∣ β,γ,α,Σ) ×P(Σ)×P(β)×P(γ),

where we have assumed that unknown parameters *β*, *γ*, and Σ are a priori independent and *α* depend only on Σ. MCMC methods are used to sample from the posterior distributions of the unknown parameters. We have used the WinBug software which uses the Gibbs sampling to form the posterior distribution for each unknown parameter by drawing samples from their full conditional distributions. 

 Our primary interest in modeling sedation levels is to investigate the effects of the some covariates as well as the transition from one sedation level to another as time since arrival progresses. To do this we consider five variations of the model in Table 1. The models contain combinations of terms to capture the covariate effects, the transition effects, and a random effects term to capture over dispersion in the form of between-subject variability. 

 In the model 1, given sedation level *j*, the regression effects remain constant but each individual *i* is considered as a cluster of responses over time (*t* = 1, 2,3). A random intercept term *α*_*ij*_ which is allowed to vary between individuals, given level *j*, is included in the model to account for time constant unobserved variability. In the model 2, given sedation level *j*, this model includes constant regression effects *β*_*j*_ for the covariates *X*_*it*_ as well as constant regression effects *γ*_*j*_ for the lagged response variable *Z*_*it*_. The term representing the lagged response may be useful in explaining the transition between sedation levels and absorb some of the unobserved variability between individuals. The model 3 is similar to model 2 with a random effect term *α*_*ij*_ included to capture any additional between-subject variation. In the model 4, given sedation level *j*, this model includes constant regression effects for the covariates but differs to model 2 as it also includes time-varying effects *γ*_*jt*_ for the lagged response variable, *Z*_*it*_. These effects are included to capture any change in the transition in sedation levels between the different times. The model 5 is similar to model 4 with a random effect term *α*_*ij*_ included to capture any additional between-subject variation. 

 The ability to fit complex hierarchical models using MCMC techniques presents a need for methods to compare alternative models. Standard model comparison techniques such as the Akaike Information Criterion (AIC) [20] and the Bayesian Information Criterion (BIC) [21] require the specification of the number of parameters in each model. For hierarchical models which contain random effects, the number of parameters is not generally obvious and so an alternative method of comparison is required. The Deviance Information Criterion (DIC) is a hierarchical modeling generalization of the AIC and BIC. It is particularly useful in Bayesian model selection problems where the posterior distributions of the models have been obtained by MCMC simulation. Like AIC and BIC it is an asymptotic approximation as the sample size becomes large.

 DIC was developed by [22]. The DIC statistic is a measure of model complexity and fit and is defined as

(6) $$\begin{array}{c} \text{DIC}=\;\bar{D\left(\theta \right)}\;+p_{D}, \end{array}$$

where *D*(*θ*) is the deviance given the model parameters $\theta ,\;\bar{D(\theta )}$ is the posterior mean of the deviance, $D(\bar{\theta })\;\;$is the deviance evaluated at the posterior mean *θ*, and $p_{D}=\bar{D}(\theta )-D(\bar{\theta })\;\;$is the effective number of parameters in the model. The quantities $\bar{D(\theta )}$ and $D(\bar{\theta })\;\;$are easily computed from an MCMC simulation chain.

## 3. Application to Sedation Data

A part of the data was used by [23]. They compared the effects of Midazolam, Diazepam, Luminal, and Cardiac Cocktail in terms of sedation level. Also 127 children who received MRI and CT were included in this study. Group M (*n* = 30) received Midazolam, Group D (*n* = 31) received Diazepam, Group L (*n* = 32) received Luminal, and Group C (*n* = 34) received Cardiac Cocktail. Systolic Blood pressures, Pulse rates, the number of breathe, and oxygen saturation were monitored. The other measurements, which may affect the sedation level, such as weight, disease status, test status, complication status, age, and adaptation status, were also recorded. Descriptions of predictor values used in the analysis are given in Table 2.

Models in Table 1 were constructed according to the assumption that sedation levels are distributed as a multinomial random variables with the six possible categories as in Table 3. Sedation levels were maintained in the range of Ramsey Scale from 1 to 6 for the 15th minute, 30th minute, and 60th minute. The Ramsay Sedation Scale was given in Table 3.

 The models were constructed according to the assumption that sedation levels are distributed as multinomial random variables with the six possible categories. Since there is little information available about the parameters, we choose noninformative prior distributions for the parameters. For regression parameters *β* ~ Normal(0, 10^3^) and *γ* ~ Normal(0, 10^3^), we assume that the random effects *α*_*i*_ are drawn from a multivariate normal distribution with zero mean and a variance-covariance matrix Σ. Noninformative uniform priors were determinated for the individual elements of Σ. Σ_11_ and Σ_22_ were given uniform (0, 100) priors and Σ_12_ = Σ_21_ was assigned uniform $\left(-\sqrt{\Sigma_{11}\Sigma_{22}},\;\sqrt{\Sigma_{11}\Sigma_{22}}\right)$ prior. 

 Gibbs sampler was run for 10.000 iterations with the first 1000 as burn-in. Convergence for the posterior distributions of all models was achieved. We set up five multinomial models with six possible sedation levels for each model in Table 1. Therefore 30 models were constructed. Posterior calculations were calculated for all models. As an example posterior summaries for the effect on log⁡⁡[*P*(Sed6)/*P*(Sed1)] using model 1 are represented in Table 4.

It is easy to say from Table 4 that there are associations between the response and some explanatory variables. The explanatory variable group D, weight, comp, SPS, and PUL have significant effect on log⁡⁡[*P*(Sed6)/*P*(Sed1)]. We have the similar posterior results for all thirty models. Estimated posterior means and %95 intervals for the effects of all explanatory variables in Table 2 on the log⁡⁡ of the probability of a patient being in Sed6 relative to the probability of being in Sed1 from models 1, 2, 3, 4, and 5 were obtained. They are given in Table 5. The variable Sed-lev.(*t* − 1) refers to the sedation levels of the patient in the previous time. The corresponding effect in the model is averaged over the 2 steps between times. The variable Sed-lev.(*s*, *t* − 1) refers to the previous sedation levels at time *s* for *s* = 2,3.

From Table 5, we can say that the explanatory variable group D, weight, comp, SPS, and PUL have significant effect on log⁡⁡[*P*(Sed6)/*P*(Sed1)] for models 1, 2, 3, 4, and 5. We also certainly state that there is relationship between the current sedation level and the sedation level at the previous time of measurements for all models.

For model comparisons, DIC values for all effect for each model were calculated. The DIC values were given in Table 6.

Firstly, Deviance Information Criteria (DIC) value was obtained at three times for all models with different effect. Table 6 compares the models when the deviance is obtained at three times. Model 2 and Model 4 are log models and essentially condition time 1 and model 1 explains time 1, with model 1 which explain time 1. We also calculate the DIC* at times 2 and 3. Therefore we focus on prediction of these times only. 

## 4. Conclusions

Results in Table 6 show that the DIC for model is smaller than the DIC for the other models. Model 1 which contains a random effects term for each patient and sedation level over time shows better performance than the other models. 

 Model 2, which includes a transition variable, shows the similar performance with models 3, 4, and 5. If we are concerned with the prediction of times 2 and 3 only, model comparisons results in Table 6 show that the DIC* for models 2, 4, and 5 is smaller than model 1. Models 2, 4, and 5 provide better understanding of the effect of the changes over the three waves than Model 1. For this aim, we prefer to consider models 2, 3, 4, and 5. 

 For models 4 and 5, Table 6 shows that there is a significant difference between the transitions in sedation levels for times 1 to 2, and from times 2 to 3. Therefore we may prefer models 4 and 5 to the other models for the transitions. 

 We say that an important characteristic of hierarchical models is that each parameter referring to a specific group from the corresponding parameters of the other group.

 Using Bayesian approach makes hierarchical model more flexible than classic hierarchical models. That is why they describe the data better. Bayesian hierarchical approach simplifies the interpretation and computation of the model. 

## Figures and Tables

**Table 1:** The model used in this study.

Model	
1	log⁡(*μ*_*itj*_) = *X*_*it*_^*T*^ *β*_*j*_ + *α*_*ij*_
2	log⁡(*μ*_*itj*_) = *X*_*it*_^*T*^ *β*_*j*_ + *Z*_*it*_^*T*^ *γ*_*j*_
3	log⁡(*μ*_*itj*_) = *X*_*it*_^*T*^ *β*_*j*_ + *Z*_*it*_^*T*^ *γ*_*j*_ + *α*_*ij*_
4	log⁡(*μ*_*itj*_) = *X*_*it*_^*T*^ *β*_*j*_ + *Z*_*it*_^*T*^ *γ*_*jt*_
5	log⁡(*μ*_*itj*_) = *X*_*it*_^*T*^ *β*_*j*_ + *Z*_*it*_^*T*^ *γ*_*jt*_ + *α*_*ij*_

**Table 2:** Descriptions of predictor values used in the analysis.

Predictor	Description
Group	M: Midazolam; D: Diazepam; L: Luminal; C: Cardiac Cocktail.
Age	Between 4 months old and 13 years old.
Weight	Between 6 kg and 46 kg.
Test	CT and MRI.
Sex	Male and female.
Disease	Diseased with neurological damage. Not diseased.
SBP	Systolic blood pressure.
PUL	Pulse.
OSAT	Oxygen saturation.
Comp	Complication: yes and no.

**Table 3:** Ramsay sedation scale.

Categories	Response
Sed1	Anxious or restless or both
Sed2	Cooperative, orientated, and tranquil
Sed3	Responding to commands
Sed4	Brisk response to stimulus
Sed5	Sluggish response to stimulus
Sed6	No response to stimulus

**Table 4:** Posterior summaries for the effect on log⁡[*P*(Sed6)/*P*(Sed1)] using Model 1.

Variables	Mean	Sd	MC error	%2.5	Median	%97.5	Start	Sample
Group								
C	−3,1681	4,172	0,0043	−1,121	−3,017	7,016	1000	10.000
D	**−53,215**	**1,931**	**0,0011**	**−53,131**	**−52,911**	**−50,97**	**1000**	**10.000**
L	17,312	10,171	0,0211	−21,431	16,951	23,71	1000	10.000
Age	0,017	0,021	0,0001	−0,015	0,021	0,038	1000	10.000
Sex [male]	−0,312	0,295	0,003	−0,773	−0,331	0,121	1000	10.000
Disease [1]	−0,215	0,211	0,015	−0,328	−0,231	0,174	1000	10.000
Weight	**−0,1311**	**0,0111**	**0,001**	**−0,151**	**−0,1417**	**−0,1317**	**1000**	**10.000**
Comp (yes)	**0,087**	**0,0095**	**0,002**	**0,065**	**0,081**	**0,093**	**1000**	**10.000**
Test (1)	0,137	0,131	0,021	−0,021	0,136	0,141	1000	10.000
Sps	**−0,016**	**0,003**	**0,003**	**−0,6171**	**−0,015**	**−0,0139**	**1000**	**10.000**
Pul	**−0,0121**	**0,002**	**0,0001**	**−0,0729**	**−0,012**	**−0,011**	**1000**	**10.000**
OSAT	−0,117	0,011	0,0002	−0,018	−0,013	0,011	1000	10.000

**Table 5:** Estimated posterior means and %95 intervals.

Variables	Model 1	Model 2	Model 3	Model 4	Model 5
Group					
C	−3,017 (−4,121; 7,016)	−2,981 (−3,115; 6,812)	−2,17 (−2,29; 5,61)	−4,21 (−5,51; 9,82)	−3,81 (−6,61; 3,43)
D	**−52,911 (−53,131; −50,97)**	**−48,17 (−52,16; −46,13)**	**−37,4 (−48,1; −29,8)**	**−41,4 (−53,1; −26,1)**	**−43,5 (−51,4; −31,5)**
L	16,931 (−21,431; 23,71)	15,81 (−21,03; 21,74)	10,71 (−5,03; 15,2)	13,15 (−4,81; 27,16)	17,51 (−3,85; 21,12)
Age	0,021 (−0,025; 0,039)	0,018 (−0,074; 0,041)	0,17 (−0,43; 0,48)	0,07 (−0,29; 0,61)	0,11 (−0,41; 0,23)
Sex	−0,331 (−0,773; 0,121)	−0,365 (−0,443; 0,141)	−0,91 (−1,21; 0,78)	−0,45 (−1,54; 1,131)	−0,71 (−1,13; 1,45)
Disease	−0,231 (−0,328; 0,174)	−0,261 (−0,317; 0,161)	−0,98 (−1,67; 0,71)	−0,631 (−1,27; 0,617)	−0,73 (−1,11; 0,62)
Weight	−0,1417 (−0,151; 0,1317)	−0,1321 (−0,1617; −0,117)	−0,67 (−0,87; −0,25)	−0,24 (−0,43; 0,12)	−0,34 (−0,84; −0,17)
Comp	**0,081 (0,065; 0,093)**	**0,076 (0,051; 0,113)**	**0,162 (0,101; 0,312)**	**0,151 (0,09; 0,27)**	**0,101 (0,06; 0,17)**
Test	0,136 (−0,221; 0,141)	0,121 (−0,114; 0,151)	0,671 (−1,21; 0,83)	0,541 (−1,51; 1,19)	0,337 (−1,17; 0,98)
Sps	**−0,015 (−0,0171; −0,0139)**	**−0,013 (−0,031; −0,009)**	**−0,065 (−1,11; −0,03)**	**−0,047 (−1,76; −0,04)**	**−0,018 (−0,29; −0,03)**
Pul	**−0,012 (−0,0729; −0,011)**	**−0,015 (−0,021; −0,008)**	**−0,023 (−0,045; −0,012)**	**−0,014 (−0,21; −0,04)**	**−0,091 (−1,21; −0,02)**
OSAT	**−0,013 (−0,018; 0,011)**	**−0,021 (−0,033; 0,016)**	**−0,156 (−0,211; 0,06)**	**−0,11 (−0,35; −0,04)**	**−0,17 (−0,29; −0,13)**
Sed-level (1, *t* − 1) [Sed1]					
Sed2		**5,43 (3,98; 6,83)**	**4,91 (3,71; 5,61)**		
Sed3		**5,65 (2,68; 7,26)**	**4,73 (3,86; 5,12)**		
Sed4		**5,21 (3,12; 5,93)**	**5,13 (4,67; 5,37)**		
Sed5		**5,85 (3,29; 6,81)**	**4,88 (4,21; 5,17)**		
Sed6		**5,43 (4,71; 4,81)**	**5,13 (4,91; 5,61)**		
Sed-level (2, *t* − 1) [Sed1]					
Sed2				**5,13 (4,81; 5,61)**	**5,41 (4,71; 5,91)**
Sed3				**5,29 (4,61; 5,79)**	**5,16 (4,51; 5,56)**
Sed4				**5,41 (4,71; 4,81)**	**5,23 (4,91; 5,61)**
Sed5				**5,35 (4,91; 5,81)**	**5,44 (5,01; 5,96)**
Sed6				**5,25 (4,12; 5,93)**	**5,23 (4,67; 5,37)**
Sed-level (3, *t* − 1) [Sed1]					
Sed2				**5,29 (4,87; 5,65)**	**5,96 (5,26; 6,36)**
Sed3				**5,17 (4,67; 5,81)**	**5,91 (5,31; 6,31)**
Sed4				**5,27 (4,71; 5,67)**	**5,77 (5,27; 6,28)**
Sed5				**5,13 (4,55; 5,87)**	**5,85 (5,11; 6,51)**
Sed6				**5,31 (4,71; 5,81)**	**5,33 (4,91; 5,61)**

**Table 6:** The DIC values for model comparisons.

Effect	Model	DIC	DIC*
log⁡[*P*(Sed6)/*P*(Sed1)]	1	28,71	72,17
	2	30,81	63,15
	3	30,76	62,17
	4	30,14	63,28
	5	30,95	63,17

log⁡[*P*(Sed5)/*P*(Sed1)]	1	26,16	74,85
	2	29,64	67,61
	3	29,71	66,75
	4	29,67	66,81
	5	29,17	66,37

log⁡[*P*(Sed4)/*P*(Sed1)]	1	26,73	75,11
	2	28,95	67,91
	3	28,72	67,54
	4	28,67	67,17
	5	28,81	67,18

log⁡[*P*(Sed3)/*P*(Sed1)]	1	25,03	73,71
	2	28,75	67,55
	3	28,19	68,01
	4	28,63	67,85
	5	28,85	67,47

log⁡[*P*(Sed2)/*P*(Sed1)]	1	26,25	72,19
	2	29,37	65,16
	3	29,85	64,18
	4	29,67	65,93
	5	29,17	65,41

*Shows the DIC value at times 2 and 3.
